# Control of gamma vs beta competition in olfactory bulb by the balance between sensory input and centrifugal feedback control

**DOI:** 10.1186/1471-2202-16-S1-F3

**Published:** 2015-12-18

**Authors:** François David, Emmanuelle Courtiol, Nathalie Buonviso, Nicolas Fourcaud-Trocmé

**Affiliations:** 1 Lyon Neuroscience Research Center, CNRS UMR 5292, INSERM U1028, Université Claude Bernard, Lyon, France; 2 Emotional Brain Institute, Nathan Kline Institute for Psychiatric Research and the Department of Child and Adolescent Psychiatry New York University Langone Medical Center, New York, NY, USA

## 

Gamma (40-80Hz) and beta (15-40Hz) oscillations and their associated neuronal assemblies are key features of neuronal sensory processing. However, the mechanisms involved in either their interaction and/or the switch between these different regimes in most sensory systems remain misunderstood. The mammalian olfactory bulb (OB) expresses both gamma and beta oscillations, which appear to be mutually exclusive, and a slower one related to respiration (2-10Hz). Gamma oscillations have been linked to odorant physical properties (quality, intensity) while beta oscillations are strongly increased by odor experience (for reviews see [1,2]). Importantly, the occurrence pattern of these two fast alternating oscillations is intermingled with the respiratory slow rhythm which provides a window for odor discrimination. Based on in vivo recordings and biophysical modeling of the mammalian olfactory bulb (OB), we explored how OB internal dynamics and the balance between sensory and centrifugal inputs control the occurrence and alternation of OB gamma and beta oscillations over a respiratory cycle.

In the OB, fast oscillations originate in the dendrodendritic interaction between excitatory mitral cells (MCs) and inhibitory granule cells (GCs). Experimental evidence has shown that GC dendritic arbor can operate in two modes: a local mode which effectively allows a weak inhibition between MCs without requiring GC spikes, and a global mode which induces a strong inhibition of MCs following GC spikes. We implemented these two inhibitory mechanisms in a parsimonious and flexible OB model based on generalized integrate-and-fire models. In the granule non-spiking regime, the weak inhibition can sustain OB oscillation in the gamma frequency range with characteristics of an auto-entrainment process [3]. In contrast, in the granular spiking regime, MCs sufficiently excite the GCs such that the latter discharge and induce a strong inhibitory input which silences the MC population and generate beta oscillations, similarly to the PING regime [4]. Intrinsic properties of each type of oscillation are remarkably stable regarding most of tested network parameters. However their occurrence depends strongly on OB network sensory and centrifugal inputs (onto MCs and GCs respectively). In particular, sensory activation of MCs must be strong enough for the emergence of gamma oscillations, while sufficient centrifugal activation of GCs, to allow them to spike, is necessary to generate beta. Based on novel experimental data in anesthetized rat, we show that both inputs are slowly modulated by the respiratory rhythm but phase shifted by about a quarter cycle. In our model, this phase shift can account for the gamma-beta alternation observed in vivo. Finally, additional tests show that the model captures accurately the competition between gamma and beta oscillations when sensory or centrifugal inputs are modulated such as in different natural conditions involving odor characteristics (odor intensity) and behavior (odor experience, active sniffing).

Overall the model approaches very closely OB dynamics observed in vivo, and can thus be used to interpret present and future experiments.
